# The British Journals, &c.: Medical Jurisprudence and Toxicology

**Published:** 1842-07

**Authors:** 


### MEDICAL JURISPRUDENCE AND TOXICOLOGY.

REMARKABLE CASE OF SUICIDE. A woman aged 29, and previously in robust health, was found dead in her apartment. In accordance with the provisions of the anatomy act, her body was received by Dr. Handyside for dissection, and was by him, along with an assistant, carefully examined. Nothing was found sufficient to account for the death; and Dr. Handyside believing her to have died of "simple apoplexy," had the lips sewed together, proposing to reserve the body for his lectures. About two months after, while about to demonstrate on this subject, the muscular structure of the pharynx, Dr. Handyside introduced his finger into the back part of the mouth, in order to stuff that cavity with hair; but found this space preoccupied by a dense foreign substance, apparently round in form, and impacted between the roof of the tongue and the soft palate, so very firmly, as to have cut off the supply of air into the lungs during life, by forcibly closing the mouth and posterior nares. The materials of this plug consisted of portions of soft cotton, called spindle ends. Some of this cotton, the woman had untwisted and rolled up very closely, coiling over it two strips of flannel. Finally, she had fastened these materials together with a large rough pin. On examination, the soft palate presented to view, on the left side, (the one corresponding to that portion of the plug, where the rough head of the pin projected,) a small but deep laceration, and which notwithstanding the long interval, was surrounded by a circumscribed patch of ecchymosis, still of a vermilion hue. The right side of the soft palate, the anterior fourth of the tongue and the hard palate opposite to it, the epiglottis, and the arytenoid cartilages, which appeared to have been violently separated by the last expiration, also exhibited ecchymoses.

The author infers from the above case, the necessity in cases of medico-legal enquiry, of paying attention to the natural apertures. He also calls attention to the length of time that the appearance of recent ecchymosis lasted.

The author cites a somewhat similar case, described by Dr. Wagner, as having occurred in Berlin. It was that of a criminal who was found dead in his cell. Another case also occurred about five years ago, in Edinburgh. [Qy. might not this have been a case of murder in place of suicide?]—Dr. Handyside, *Edin. Med. and Surg. Journ.* No. 151. *April* 1, 1842.

---

Detection of Arsenic in Complicated Liquids. After remarking that Marsh's test, from its very perfection of delicacy, is not easily manageable in the hands of any not thoroughly conversant with chemical research, the author proposes the following plan, in the employment of which, not a single instance of failure has occurred.

When dilute sulphuric acid is boiled with the greater number of the substances used for food, and which are likely to become the objects of chemico-legal investigations, the invariable effect is the acquisition, by such substances, of a great degree of thinness and limpidity, partly by the conversion of the starch and mucilaginous matters they contain, into dextrin and sugar, and partly also by the coagulation of the albumen and casein present. This is very strikingly shown by boiling, for a few minutes, a quantity of thick gruel with a little dilute oil of vitriol; the mixture becomes quite thin, and runs through a paper filter, almost as freely as pure water, a coagulum of azotized substance being left behind. The same thing happens with milk and beer, and many other complicated liquids; they become as thin as water, and filter quite easily. A clear solution being thus got, a stream of sulphuretted hydrogen is, when cold, passed through it, the liquid boiled for a few seconds, and then passed through a small filter, and the orpiment washed. In this state it is seldom pure enough for advantageous reduction, even when its colour is bright; it is better to dissolve it in aqua regia, to evaporate gently to dryness, take up the residue with water, and again to precipitate with sulphuretted hydrogen, wash, dry, and reduce with black flux. The second precipitation by sulphuretted hydrogen requires some care, as the arsenic is often not at first thrown down from being in the state of arsenic acid; it is proper, after passing the gas some time, to heat the liquid to its boiling point, suffer it to cool, send through it an additional portion of gas, and again boil; by which all the orpiment falls down at once, and is easily collected on a little filter. The solution should also be acidulated with a little hydrochloric acid. When such a substance as soup is to be examined, rich in gelatine, it is better to get rid of that body by the aid of an infusion of gall-nuts before proceeding to the treatment by sulphuric acid.—George Fownes, Esq., *Pharmaceutical Journal,* No. x. *April* 1, 1842.

